# Traditional treatment of human and animal salmonelloses in Southern Benin: Knowledge of farmers and traditherapists

**DOI:** 10.14202/vetworld.2017.580-592

**Published:** 2017-06-03

**Authors:** T. V. Dougnon, E. Déguénon, L. Fah, B. Lègba, Y. M. G. Hounmanou, J. Agbankpè, A. Amadou, H. Koudokpon, K. Fabiyi, A. Aniambossou, P. Assogba, E. Hounsa, M. de Souza, F. Avlessi, T. J. Dougnon, F. Gbaguidi, M. Boko, H. S. Bankolé, L. Baba-Moussa

**Affiliations:** 1 Research Laboratory in Applied Biology, Polytechnic School of Abomey-Calavi, University of Abomey-Calavi, 01 PO Box 2009 Cotonou, Benin; 2 Laboratory of Hygiene, Sanitation, Toxicology and Environmental Health, Interfacultary Center of Training and Research in Environment for Sustainable Development, University of Abomey-Calavi, 01 PO Box 1463 Cotonou, Benin; 3 Laboratory of Biology and Molecular Typing in Microbiology, Faculty of Sciences and Techniques, University of Abomey-Calavi, 05 PO Box 1604 Cotonou, Benin; 4 Laboratory of Training and Research in Applied Chemistry, Polytechnic School of Abomey-Calavi, University of Abomey-Calavi, 01 PO Box 2009 Cotonou, Benin; 5 National Laboratory of Pharmacognosy, Beninese Center for Scientific and Technical Research, 01 PO Box 06 Oganla, Porto-Novo, Benin

**Keywords:** farmers, medicinal plants, salmonellosis, Southern Benin, traditherapists

## Abstract

**Aim::**

This study aimed to report medicinal plants that are likely to be used in the control of salmonellosis.

**Materials and Methods::**

A cross-sectional study was conducted in Southern Benin. Semi-structured questionnaires were administered to 150 farmers and 100 traditional therapists in seven high municipalities. This step helped to collect plants that are used in the treatment of animal salmonellosis and typhoid fever in human.

**Results::**

The results revealed a low level of use of medicinal plants among breeders who prefer antibiotics such as oxytetracycline (53.55%), tylosine + sulfadimerazine (15.30%), and alphaceryl (19.13%). However, plants such as *Moringa oleifera* (leaves), *Carica papaya* (leaves and seeds), and *Vernonia amygdalina* (leaves) were mostly used by some farmers. From traditional therapists, 57 plant species of 32 families were identified as typhoid fever cures; among which Leguminosae, Asteraceae, and Euphorbiaceae were predominant. *Persea americana* (22.72%), *V. amygdalina* (7.57%), and *Corchorus olitorius* (7.57%) were the most cited by traditherapists for the treatment of typhoid fever in human.

**Conclusion::**

This study provides a database for further studies on the *in vitro* and *in vivo* efficacy of Benin plant species on *Salmonella* *spp*. These evaluations will guarantee the availability of new therapeutic solutions for populations.

## Introduction

Animal husbandry is one of the main activities undertaken in developing countries for livelihood. It contributes greatly to the world economy. However, the performances of the livestock sector can be hindered by a number of factors including infectious diseases such as salmonelloses [1]. In developing countries such as Benin, enteric infections and foodborne diseases constitute a major preoccupation of public health because of their incidence and severity. Foodborne diseases cause more than 17 million deaths per year worldwide, and more than half occurs in Africa [2]. Animals have been reported to be the main sources of transmission of *Salmonella* spp. to human. The poor hygiene level of the livestock systems in West Africa can encourage the introduction of pathogenic germs in farms [1]. Serotypes of *Salmonella* that cause minor salmonelloses are part of the important pathogenic foodborne bacteria, with a large range of hosts, including animals and human. *Salmonella* Enterica is one of the main causative agents of foodborne diseases (11%), hospitalization (35%), and death (28%) in the United States of America [3]. Moreover, most strains of *Salmonella* species, like *S. Typhimurium*, were proven to be resistant to many antibiotics [4]. It is universally admitted that the emergence of antimicrobial resistance, in particular, multidrug resistance (MDR) of *Salmonella* strains to ampicillin, chloramphenicol and cotrimoxazole, has complicated the treatment and management of salmonellosis [5]. In spite of the increasing negative impact of salmonelloses on public health, limited information is available regarding the prevalence, the antibiotics susceptibility and the mechanism of MDR of these strains in Benin. Actually, the control of the evolution of drug-resistant strains of *Salmonella* at animals’ level is a key to preserving the transmission toward humans who are big consumers of meat.

Typhoid fever, a potentially life-threatening gastrointestinal infection, is caused by a non-spore bearing bacilli called *Salmonella* Enterica serovar Typhi (*S. Typhi*) [6]. This bacterium is transmitted by faecal-oral route with the organism gaining entry into the body through the intestinal mucosa [7]. Advances in public health strategies, technology, and hygiene have led to the eradication of typhoid fever from the developed world, but since the 1800s, typhoid fever has remained an endemic disease in many developing countries [8,9].

This is worrying because of the high morbidity and mortality associated with typhoid fever [10], particularly in children under 5 years old [11,12] and the emergence of MDR *S. Typhi* [9,13-15]. The sources of infection vary but the most common mode of transmission is by ingesting an infective dose of *S. Typhi* through contaminated food or water. The true global disease burden of typhoid fever is difficult to be estimated as few established surveillance systems on typhoid fever exist in developing countries [9].

For example, in Africa, the overall burden of typhoid fever remains largely unknown, mainly because facilities capable of performing the blood culture tests essential for diagnosis are absent from many regions [16].

Untreated typhoid fever can lead to gruelling complications such as gastrointestinal hemorrhage, hepatomegaly, anorexia, diarrhea, toxicity, encephalopathy, myocarditis, and disseminated intravascular coagulation [17]. For decades, antibiotics such as chloramphenicol, ampicillin, and cotrimoxazole were the mainstay of typhoid treatment [18]. However, the widespread emergence of MDR *S. Typhi* necessitated the search for other therapeutic alternatives such as the fluoroquinolones, third generation cephalosporins, and azalides [19]. Fluoroquinolones are, however, restricted from routine use in children and quinolone–resistant *S. Typhi* strains have been documented [20-22]. Ceftriaxone is highly effective against *S*. *Typhi* but parenteral administration limits its usage [20]. Azithromycin is however very effective against *S. Typhi* [23]. These drugs are very expensive and less affordable to a majority of individuals in developing countries such as Benin, hence a greater proportion of the population resort to the use of herbal medicines. It is estimated that about 80 % of the world’s population rely on traditional medicine, particularly herbal medicine for primary health care [2,24,25].

Benin is a land of immense biodiversity and a hub of very potent and efficacious medicinal plants which are involved either in the holistic treatment of diseases or the alleviation of symptoms. An impressive number of plants have been used traditionally in the treatment of typhoid fever in Africa since immemorial times [26].

This survey was therefore carried out to investigate plants used by breeders to treat salmonelloses in animals and those used by traditional healers to treat typhoid fever in human. The final goal is the development of new, cost-effective and easily accessible medicines against typhoid fever in Benin but also to overcome MDR of *Salmonella* spp.

## Materials and Methods

### Ethical approval

The study was conducted among Beninese individuals. Each target was maintained in relation to the objectives of the study and a written consent form was signed. These targets were free to participate or not to participate in the study.

### Study area and materials

The study was conducted in Southern Benin located between 6° 25N and 7° 30’N, covering a surface of 17109 km^2^. The climate is of sub-equatorial type, characterized by a bimodal rainfall regime with two rainy seasons alternated by two dry seasons. The average annual temperature is 28°C, and the air humidity varies between 69% and 97% [27]. The most dominant soils are ferralitic soils on clayey sediments, hydromorphic soils in the valleys, shallows and alluvial plains, vertisoils in the depression of Lama, and tropical eutrophic brown soils [28]. Phytogeographically, Southern Benin is subdivided into four districts: Coastal, Pobè, Ouémé Valley, and Plateau [29]. It belongs to the Guineo-Congolese zone that comprises mosaic humid and dense islets of forests, savannahs, prairies, mangrove swamps, and fallows. The present ethnopharmacological survey was conducted in 7 Municipals of Southern Benin, namely, Porto-Novo, Dangbo, Cotonou, Abomey-Calavi, Sèmè, and Adjarra and Ouidah (Table-1). These municipals were chosen based on the presence of many traditherapists and farmers revealed by the previous studies [30]. The materials used were questionnaires for interviews, digital camera, voice recorder, plastic bags, sticky tape, and marker pens.

**Table-1:** Townships covered by the survey.

Nationals departments of Southern Benin	Investigated areas
Atlantique	Abomey-Calavi
Littoral	Cotonou, Ouidah
Plateau	Sèmè
Ouémé	Porto-Novo, Dangbo, Adjarra

### Sampling

The study enrolled 50 pig farmers, 50 chicken farmers, and 50 cattle keepers randomly selected from the list of farmers available in Southern Benin. These farmers were interviewed within a semi-structured questionnaire. The selection of the three types of animals (pigs, chicken and cattle) was justified by their predominance in the food habit of the concerned study population. For traditherapists, a total of 100 people were enrolled in the study. This was based on stratified random sampling.

### Sampling procedure

One-on-one interviews were conducted using semi-structured questionnaires for farmers and traditional healers.

#### Interview with farmers

A questionnaire was designed and administered to probe information on botanical, ethnobotanical, and ethnoveterinary potentials of the traditional recipes used against salmonelloses in animals. Further information collected based on the questionnaire was the profile of the interviewed farmer, the local names of the plants used to treat or to prevent salmonellosis and the recipes related to these plants.

#### Interviews with traditherapists

A questionnaire was designed to collect information related to botanical, ethnobotanical, and ethnopharmacological potentials of the recipes used against typhoid fever. The investigation starts by a preparatory phase during which meetings were organized with the traditional healers. The collected information was basically about the profile of the investigated therapist and the ethnopharmacological data of the plants used to treat or to prevent typhoid fever.

For the two surveys, we were focused on the local names and the parts of plants that are used, as well as the therapeutic indications, all possible recipes, the mode of administration, and the potential side effects.

### Identification of plant species

The plants mentioned in the local languages Fon, Goun and Mina were compared with other references such as Contribution to ethnobotanical and floristic studies in Populary Republic of Benin [31]; Flora of Benin: Names of plants in Beninese National languages [32] and the analytic flora of Benin [27].

### Literature review

A scientific literature review was performed on plants potentially active in the treatment of salmonellosis to make an inventory of the already existing data on the plants revealed by this study. It was also meant to record further plants reported in the literature for their anti-*Salmonella* properties but not mentioned in the present study. This was a complete desk study searching through online scientific databases, specialized works, and publications. The number of references documented, the relevance, and the repetition of the information was considered. A large number of references on a plant frequently cited from different countries demonstrate a certain medicinal interest of that particular plant. The information was judged significant when reported in several references.

### Statistical analyses

The collected data were encoded and recorded in an Excel database. Descriptive statistics were performed using SPSS 20.

## Results

### Endogenous knowledge of farmers related to plants used in the treatment of animal salmonellosis

#### Sociocultural characteristics of farmers included in the survey

A total of 150 farmers were interviewed including 96% male and 4% female farmers unequally distributed in the townships of Abomey-Calavi (76%), Cotonou (2,67%), Ouidah (6,67%), Porto-Novo (6,67%), Sèmè-Kpodji (4,67%), Dangbo (1,33%), and Adjarra (2%). The highest number of investigated farmers was found in Abomey-Calavi (76%) whereas Dangbo harbors the lowest number of enrolled farmers (1.3%).

Livestock practices are one of the dominant activities in Southern Benin. The study showed that more men are involved in these activities than women. Respondents belong to diverse ethnic groups mainly Fon and Goun but some were Mina, Yoruba, and Bariba.

Animal production in the study area is a lucrative activity that commonly uses domestic workforce but sometimes coupled with salaried employers.

Most animals were raised in confinement except cattle that were kept on free grazing but under the control of Peulh herders who are mostly illiterate and inherited the animals from their parents.

Several wards of Abomey-Calavi, Cotonou, Ouidah, Porto-Novo, Sèmè-Kpodji, Dangbo, and Adjarra were visited to meet farmers who participated in this study. Abomey-Calavi had the highest proportion of farmers. The study also revealed that the investigated farmers have other activities and were classified into four categories: Exclusive animal producers (88%) followed by 5.33% of crop producers and 4% traders then 2.67% of veterinarians.

The study revealed that 22.67% of the farmers have high-level education, 48.67% have secondary education, 16.67% have primary education, and 12.00% were illiterate. About 55.55% of farmers who went to University had professional training on animal husbandry, and 33% of all respondents had an informal training for animal farming while 6.66% became breeders just by inheritance. The youngest breeder was 23 years and the oldest 62 years old.

Table-2 shows the distribution of farmers based on their age, size of household, professional experience, and herd size.

**Table-2:** Distribution of farmers based on their age, size of household, professional experience and herd size.

Variable	Maximum	Minimum	Mean	Standard error
Age	62	20	37.59	0.89
Household size	12	2	4.49	0.16
Professional experience	27	1	7.98	0.52
Herd size	4500	10	520.01	65.78

#### Knowledge of breeders on plants used in the treatment of animal salmonellosis

Out of the 150 farmers enrolled in the study, only 36% mentioned plants that are useful as anti-­*Salmonella*. In majority of cases, this knowledge was acquired from another farmer. They do not use the plants in the treatment of their animals because there is no evidence of effectiveness of these plants.

#### Plants used by selected farmers against Salmonellosis

As displayed in Table-3 [33-40], *Moringa oleifera* (leaves), *Carica papaya* (leaves and seeds), and *Vernonia amygdalina* (leaves) were the most cited plants used by farmers to treat salmonellosis in slaughter animals. The same table also shows that these plants are used either dried or macerated. They are given to the animals orally in feed or water (Table-3).

**Table-3:** Diversity of plants species used as anti-*Salmonella* in animals.

Scientific name	Vernacular name (Fongbe)	Used part	Frequency of citation	Mode of preparation	Mode of administration	Citation in literature
*Moringa oleifera*	Kpatiman wini-wini	Leaves	30	Dried	Oral in feed	[33,34]
*Ocimum gratissimum*	Tchayo	Leaves	04	Macerated	Oral in water	[35]
*Carica papaya*	Kpin	Leaves and seeds	14	Dried	Oral in feed	[36,37]
*Cajanus cajan*	Klouékoun	Leaves	4	Dried	Oral in feed	[38]
*Vernonia amygdalina*	Amanvivè	Leaves	8	Macerated	Oral in water	[39,40]
*Manihot esculenta*	Fingnin man	Leaves	4	Macerated	Oral in water	[37]

#### Antibiotics used in animals

All the investigated farmers use synthetic antibiotics. The most cited antibiotics are classified in Table-4. These antibiotics are used as preventive and curative drugs. However, antibiotics are systematically used when animals are sick. Oxytetracycline was the most cited antibiotic (53.55%), followed by tylosine + sulfadimerazine and alphaceryl with citation frequencies of 15.30% and 19.13%, respectively.

**Table-4:** Antibiotics used by farmers for the treatment of their animals.

Antibiotics	Number of citations	Proportion
Oxytetracycline	98	53.55
Tylosine+sulfadimerazine	28	15.30
Alphaceryl	35	19.13
Flumec	2	1.09
Tetracolivit	8	4.37
Penistrepto	2	1.09
Azemite	2	1.09
Colideto	2	1.09
Biocalin	2	1.09
Amprolium	2	1.09
Alphamet	2	1.09
Total	183	

#### Endogenous knowledge of traditherapists related to plants used for the treatment of human salmonellosis

A total of 100 traditherapists were enrolled including 97% men and 3% women, from 7 municipalities of Southern Benin: Abomey-Calavi, Cotonou, Ouidah, Porto-Novo, Sèmè-Kpodji, Dangbo, and Adjarra. The majority of these respondents were found in Porto-Novo (18%), while Cotonou had the lowest number of traditherapists (10%) (Table-5).

**Table-5:** Distribution of respondents by townships.

Townships	Number	Proportion (%)
Abomey-Calavi	13	13.00
Cotonou	10	10.00
Ouidah	15	15.00
Porto-Novo	18	18.00
Seme-Kpodji	13	13.00
Dangbo	15	15.00
Adjara	16	16.00
Total	100	100.00

About 78% of the traditherapists were illiterate. The remaining had primary, secondary, or university education. They belong to 4 ethnic groups of Benin: Fon (42%), Goun (32%), Wémè (15%), and Yoruba (11%). Most of them were Fon (42%) whereas the minority was Yoruba.

#### Knowledge of traditherapists about plants that serve as anti-Salmonella in human

The most respondents were not familiar with the word “Salmonellosis.” Symptoms of diarrheal infections and typhoid fever were therefore used to describe salmonelloses. At least one recipe was recorded per traditherapist. The majority of them hesitated to provide the requested information.

The listed recipes contain 57 plant species of 32 families of which the most represented ones were Asteraceae, Leguminosae, and Euphorbiaceae (Table-6). The most cited species in the recipes were: *Persea americana* (22.72%), *Vernonia amygdalina* (7.57%), *Corchorus olitorius* (7.57%) (Table-7). Plants mentioned in the present survey are used either alone or in association with other plants. However, some recipes contain non-plant materials such as alcohol, palm oil, akassa (maize dough), and cow milk. The most used plant organs are the leaves, but also the roots, the bark or the whole plant. Most recipes are prepared as decoctions, but some are macerated in alcohol or water. All recipes are administered orally.

**Table-6:** Botanical families represented in the medicinal plants cited in the treatment of salmonellosis.

Botanical family	Proportions
Lauraceae	1.75
Annonaceae	1.75
Apocynaceae	5.26
Arecaceae	5.26
Asteraceae	7.02
Bignoniaceae	3.51
Bromeliaceae	1.75
Capparidaceae	1.75
Caricaceae	1.75
Clusiaceae	1.75
Combretaceae	1.75
Connaraceae	3.51
Cucurbitaceae	3.51
Dracaenaceae	1.75
Euphorbiaceae	7.02
Fabaceae	5.26
Lamiaceae	5.26
Leguminosae-Caesalpinioideae	5.26
Leguminosae-Mimosoideae	1.75
Leguminosae-Papilionoideae	3.51
Liliaceae	1.75
Meliaceae	1.75
Moraceae	1.75
Moringaceae	1.75
Myrtaceae	3.51
Papaveraceae	1.75
Phyllanthaceae	1.75
Piperaceae	1.75
Poaceae	3.51
Rutaceae	1.75
Salicaceae	1.75
Solanaceae	1.75
Sterculiaceae	3.51
Malvaceae	175
Total	100

**Table-7:** Plants used by traditherapists in Southern Benin against human salmonellosis.

N	Species	Family	Vernacular name	Organs used	Frequency of citation	Mode of utilization (alone or in association)	Previous references
1	*Cassytha filiformis* L.	Lauraceae	Agbégbékan	Whole plant	1.51%	Alone or in association	
2	*Psidium guayava* L.	Myrtaceae	Kinkoun man	Leaves and roots	6.06	In association	
3	*Jatropha gossypifolia*	Euphorbiaceae	Yonkpotin vovo man	Leaves	3.03	In association	
4	*Cola nitida*	Sterculiaceae	Gba’n dja	Fruits	1.51	Alone	
5	*Vernonia amygdalina*	Asteraceae	Aman vivè	Leaves	7.57	Alone or in association	Eth: [41] Pharm: [39]
6	*Crateva adansonii* DC.	Capparidaceae	Hontonzouzoin	Leaves	1.51	Alone or in association	Eth: [41] Pharm: [39]
7	*Xylopia aethiopica*	Apocynaceae	Kpédjrékoun	Fruits	4.54	In association	Pharm: [42]
8	*Caesalpinia pulcherrima*	Leguminosae-Caesalpinioideae	Orgueil de chine	Whole plant	4.54	Alone or in association	
9	*Annona muricata*	Annonaceae	Chap chap man	Leaves	4.54	Alone	
10	*Citrus limon*	Rutaceae	Cléman	Leaves	4.54	In association	
11	*Persea americana*	Lauraceae	Avocatier	Leaves	22.72	In association	
12	*Cocos nucifera*	Arecaceae	Agonkè	Leaves	1.51	In association	
13	*Carica papaya*	Caricaceae	Papaye fruit	Roots	1.51	In association	
14	*Khaya senegalensis*	Meliacea	Caicédra	Unripe fruit	1.51	In association	
15	*Acanthospermum hispidum* DC.	Asteraceae	Ahanglon	Whole plant or bark	3.03	Alone or in association	
16	*Senna siamea* (Lam.) H.S. Irwin & Barneby.	Leguminosae- Caesalpinioideae	Kassia	Whole plant	1.51	In association	
17	*Eugenia Caryophyllata*	Myrtaceae	Atikin gba do ta	Whole plant	4.54	In association	
18	*Allium sativum*	Liliaceae	Ail	Fruits	1.51	In association	
19	*Corchorus olitorius* L.	Malvaceae	Crincrin	Leaves	7.57	In association	
20	*Abrus precatorius* L.	Fabaceae	Viviman	Whole plant	3.03	In association	
21	*Croton zambesicus* Muell.	Euphorbiaceae	Djélélé	Whole plant	3.03	In association	
22	*Cajanus cajan* (L.) Millsp.	Fabaceae	Klouékounman	Leaves	3.03	Alone or in association	
23	*Kigelia africana* (Lam.) Benth	Bignoniaceae	Gnanblikpo	Leaves	3.03	Alone or in association	
24	*Momordica charantia* L.	Cucurbitaceae	Gninsikin	Leaves	3.03	In association	
25	*Garcinia cola* Heckel.	Clusiaceae	Ahowé	Fruits	1.51	In association	
26	C*ucumis metuliferus*	Curcubitaceae	Gbohounon	Fruits	3.03	Alone or in association	
27	*Piper guineense* Schumach. & Thonn.	Piperaceae	Linlinkoun	Seeds	1.53	In association	
28	*Moringa Oléifera*	Moringaceae	Yovo kpatin ou kpatinma wini-wini	Leaves	3.03	Alone	
29	*Newbouldia laevis*	Bignoniaceae	Déssréman	Leaves	4.54	In association	
30	*Elaeis guineensis* Jacq.	Arecaceae	Déman	Leaves	1.51	In association	
31	*Ocimum gratissimum* L.	Lamiaceae	Tchiayo	Leaves	1.51	In association	Eth: [34,41] Pharm: [35,39,43]
32	*Cocos nucifera*	Arecaceae	Agonkèssin	Coconut water	1.51	In association	
33	*Catharanthus roseus*	Apocynaceae	Bonjourbonsoirdo	Whole plant	1.51	In association	
34	*Haematoxylum campechianum* L.	Leguminosae- Caesalpinioideae	Campècher	Leaves	1.51	In association	
35	*Cola millenii*	Sterculiaceae	Aloviaton	Leaves	1.51	Alone or in association	
36	*Cymbopogon citratus*	Poaceae	Timan	Leaves	1.51	Alone or in association	
37	*Ananas comosus*	Bromeliaceae	Ananas	Fruits	1.51	In association	
38	*Caesalpinia bonduc* (L.) Roxb.	Leguminosae- Caesalpinioideae	Adjikouinman	Leaves	3.03	Alone or in association	
39	*Phyllostachys aurea*	Poaceae	Feuille de roseau (bambou)	Leaves	3.03	Alone	
40	*Sansevieria liberica*	Dracaenaceae	Kpognan	Leaves	1.51	Alone	Eth: [44]
41	*Argemone mexicana*	Papaveraceae	Houètchégnon	Leaves	1.51	Alone	Pharm: [45]
42	*Salix babylonica*	Salicaceae	Saule pleureur (paratonere)	Leaves	1.51	Alone	
43	*Capsicum frutescens*	Solanaceae	Akaya	Fruits	1.51	In association	
44	*Capsicum frutescens*	Solanaceae	danhomé takin	Fruits	1.51	In association	
45	*Agelaea pentagyna* Lam.	Connaraceae	Ahanhlazu	Leaves and roots	1.51	In association	
46	*Rourea coccinea* (Thonn. Ex Schumach.) Benth	Connaraceae	Vikplonba	Leaves and roots	1.51	In association	
47	*Phyllanthus amarus* Schumach.&.Thonn.	Phyllantaceae	Hlenwé	Leaves	3.03	Alone or in association	
48	*Hyptis suaveolens* Poit.	Lamiaceae	Afio	Leaves	1.01	In association	
49	*Acanthospermum hispidum* DC.	Asteraceae	Ahanglon	Leaves	3.02	Alone or in association	
50	*Bridela ferruginea* Benth.	Phyllanthaceae	Housoukokwé	Leaves	1.01	In association	
51	*Combretum micraanthum*	Combretaceae	Quiqueliba	Whole plant	1.51	Alone or in association	
52	*Acacia sieberiana* DC.	Leguminosae-Mimosoideae	Aduwéman	Leaves	3.03	In association	
53	*Acanthospermum hispidum* DC.	Asteraceae	Awisagbé	Leaves	3.03	In association	
54	*Rauvolfia caffra*	Apocynaceae	Lèwé	Roots	1.51	Alone	
55	*Erythrina senegalensis*	Leguminosae-Papilionoideae	Kpacléssi	Leaves	1.51	In association	Eth: [46]
56	*Baphia nitida*	Leguminosae-Papilionoideae	Sokakpè	Fruits	1.51	In association	

#### Literature data on the cited plants and their anti-Salmonella properties

Some of the recorded plants were previously reported in the literature with their chemical composition and biological activities (Table-7) [41-46]. The absence of studies on pharmacological data of these plants is an important selection criterion for the identification of original chemical structures which are not yet described.

The literature reviews also revealed 38 medicinal plant species of which some are used traditionally to treat salmonelloses and typhoid fever. Some of these plants have been pharmacologically studied, and their anti-*Salmonella* properties already reported (Table-8) [47-57].

**Table-8:** Plants reported to have anti-*Salmonella* properties in the literature.

N	Species	Family	Vernacular name	Ethnobotanical data	Identified substances	Pharmacological data	References
1	*Ocimum basilicum*	Lamiaceae	Kesu kesu (fon)	Mycoplasmoses (pounded leaves)	Estragole, linalol, eugenol	Inhibitory activity on S. enterica	[34,35]
2	*Cissus quadrangularis*	Vitaceae	Assan (fon)	Avian Salmonellosis (maceration)		Antibacterial and antioxidant activity	[34,43,47]
3	*Salvia officinalis*	Laminaceae	Salmiya (arabe)		Rosmarinic, caffeic, chlorogenic acids; carnosol, flavonoids, essential oils (mainly thuyone and cineole)	Activity of the essential oils against *Salmonella* spp	[48-51]
4	*Schinus molle*	Anacardiaceae	Faux poivrier ou poivrier sauvage (français)			Activity of the essential oils against *Salmonella* spp	[48,49,52]
5	*Uncaria tomentosa* (Willd). DC	Rubiaceae	Griffe de chat (français)	Decoction of roots and bark as Antibiotic	Oxindol alkaloids pentacyclic oxindols oxindol alkaloids tetracyclic	Activity of the essential oils against *Salmonella* *Typhi*	[53]
6	*Blighia sapida*	Sapindaceae	Lisè (fon)	Bath of stem and leaves against typhoid fever			[44]
7	*Ficus exasperata*	Moraceae	Aholoman (fon)	Drink decoction or maceration against typhoid fever	Sterols and polyterpenes, polyphenols, alkaloids, flavonoids		
8	*Rudbeckia purpurea*	Echinaceae	Rudbeckie rouge (français)	Recommended drug against typhoid fever			[54]
9	*Echinacea angustifolia*	Echinaceae	Echinacée à feuilles étroites (français)	Recommended drug against typhoid fever			[54]
10	*Terminalia glaucescens*	Combretaceae	Idi-odan (Yoruba)	Diarrhea, tooth decay, malaria, typhoid fever, coughing, dermatosis	terpenoides, phenol derivatives and alkaloids	Antibacterial activity on *Salmonella Typhi* and *Salmonella Typhimurium*	[31,55-57]
11	*Bidens pilosa* Linn.	Asteraceae	Abèrèoloko (yoruba)	Decoction or maceration of leaves administered orally against typhoid fever			[5]
12	*Costus afer* Ker-Gawl.	Costaceae	Trétrégougou (fon)	Decoction or maceration of leaves administered orally against typhoid fever			[5]
13	*Dissotis prostrata*	Melastomataceae		Decoction or maceration of leaves administered orally against typhoid fever			[5]
14	*Enantia chlorantia*	Annonaceae		Decoction or maceration of leaves administered orally against typhoid fever			[5]
15	*Entandrophragma candollei* Harms.	Meliaceae		Decoction or maceration of leaves administered orally against typhoid fever			[5]
16	*Entandrophragma cylindricum*	Meliaceae		Decoction or maceration of leaves administered orally against typhoid fever			[5]
17	*Kalanchoe crenata* (Andrews) Haw.	Crassulaceae		Decoction or maceration of leaves administered orally against typhoid fever			[5]
18	*Picralima nitida* (Staph) Th & H.Dur	Apocynaceae		Decoction or maceration of leaves administered orally against typhoid fever			[5]
19	*Voacanga africana* Stapf	Apocynaceae		Decoction or maceration of leaves administered orally against typhoid fever			[5]
20	*Panax ginseng* C.A. Meyer	Araliaceae		Decoction of roots administered orally against typhoid fever			[5]
21	*Enantia chlorantha*	Annonaceae		Decoction of Bark against typhoid fever			[5]
22	*Cassia occidentalis*	Caesalpiniaceae	Rai daure, majanzafari (Hausa); Rere (Yoruba); Okidiagbara (Igbo)	Use of the leaves against typhoid fever			[46]
23	*Azadirachta indica* A. Juss	Meliaceae	Dogo yaro, darbejiya (Hausa); Amuka (Yoruba); Okwuruozo (Igbo)	Use of the leaves against typhoid fever			[46]
24	*Stereospermum kunthianum* Cham.	Bignoniaceae	Sansami (Hausa)	Bark against typhoid fever			[46]
25	*Cochlospermum tinctorium* A. Rich	Cochlospermaceae	Rawaya (Hausa); Rawaye (Yoruba); Nkalike, Obasi (Igbo)	Roots against typhoid fever			[46]
26	*Hygrophilia auriculata* (Schumach) Heine	Acanthaceae	Zazargiwa, Kayar rakumi (Hausa); Zanagodoye (Kanuri)	Typhoid fever			[46]
27	*Asparagus africanus*	Liliaceae	Mugamu adawa (Hausa); Aluki, Kaadan, Koobe (Yoruba)	Typhoid fever			[46]
28	*Acacia albida*	Mimosaceae	Gawo (Hausa); Karau (Kanuri); Kad’ha (Babur)	Typhoid fever			[46]
29	*Detarium microcarpum*	Caesalpiniaceae	Taura (Hausa); Ogbogbo, sedun (Yoruba); Ofo (Igbo)	Typhoid fever			[46]
30	*Gossypium herbaceum*	Malvaceae	Auduga (Hauda); Kaitan (Kanuri); Owu (Igbo); Laghosa (Yoruba)	Leaves, typhoid fever			[46]
31	*Cadaba farinosa*	capparidaceae	Bagayi (Hausa); Marra (Kanuri); Marka (Babur)	Leaves, typhoid fever			[46]
32	*Pilostigma reticulatum*	Caesalpiniaceae	Kargo, kalgo (Hausa); Kalur (Kanuri); B'ula (Babur); Abafe, Abafin (Yoruba)	Roots, typhoid fever			[46]
33	*Combretum glutinosum*	Combretaceae	Kattakara, taranniyi, farin ganya (Hausa); Kadaar (Kanuri); Shafa (Babur)	Leaves, typhoid fever			[46]
34	*Celtis integrifolia*	Ulmaceae	Zuwo (Hausa); Nguzo (Kanuri); Nguzo (Babur)	Typhoid fever			[46]
35	*Maytenus senegalensis*	Celastraceae	Bakororo, namijin Tsada (Hausa); Karau Karau (Kanuri); Soofi (Babur); Sepolotiun (Yoruba)	Typhoid fever			[46]
36	*Cordia africana*	Boraginaceae	Alulluba (Hausa); Alwa (Kanuri); Alwa (Babur)	Typhoid fever			[46]
37	*Cassia singuena*	Caesalpiniaceae	Rumfu (Hausa); Fanalewa (Kanuri); Bag'sha (Badur)	Typhoid fever			[46]

## Discussion

The objective of this study was to establish a record of medicinal plants that are used in the treatment of human and animal salmonellosis. This was based on the knowledge of farmers and traditional healers. These two strategic targets were chosen to make the survey more conclusive from human and animal health perspectives. The two investigations reported 63 plant species used in the treatment of salmonelloses in Southern Benin comprising 6 from farmers and 57 from traditherapists. They represent 2.24% of the total flora of the country that harbors 2807 medicinal plant species [27]. This specific abundance for salmonellosis is higher than those reported by Dassou [58] in the country; and lower as compared to the results of Adomou [44], Fah *et al*. [59], Dassou *et al*. [60].

The low level is attributable to the fact that this study was limited to plants used in the treatment of salmonelloses and can also be due to differences in the geographical areas where these studies were conducted. Furthermore, this study demonstrated that more men are involved in animal production than women in the study population. This is because the activity requires a lot of financial inputs, time but also a lot of physical effort. It can also be due to the fact that women prefer commercial activities in the study area. These results are comparable to those of Sacramento [61] who reported similar results among grasscutters keepers. Farmers of the study area do not use herbal medicines for the treatment of their animals mainly the poultry keepers for many reasons: To avoid messing up the prophylaxis, avoid the death of animals the drop of laying rate in layers because they have limited information about the posology.

They, therefore, prefer using synthetic antibiotics. Better results were reported by Dassou *et al*. [60] who recorded 241 medicinal plant species used in the treatment of 45 animal diseases and symptoms which include 0.90% of plants against salmonelloses. Nevertheless, most of the plants listed in that study are used against intestinal worms; as found by Ogni *et al*. [62] from farmers. Moreover, this study revealed that most traditherapists are men. This makes sense because most traditional healers and people involved in herbal medicine in Benin are men. It is rather the sale of the recipes in markets that is reserved for women. In recent times, public interest in natural remedies, mostly herbal medicine, has increased drastically not only in developing countries but also highly industrialized countries [63].

This has increased international trade in herbal medicine tremendously. Self-prescribed herbal remedies are on the ascendancy these days for the treatment of diseases such as headaches, insomnia, fever, intestinal disorders, and typhoid fever [64,65]. 57 medicinal plants of 32 families were recorded from traditional healers, as effective against salmonellosis. Results of this study are in accordance with those of Fadimu *et al*. [40] who recorded in Nigeria almost the same plant species in their ethnomedicinal survey on plants used in the treatment of typhoid fever. However, the most representative plant families in this survey were the Rutaceae, (then Asteraceae), Leguminosae, and Euphorbiaceae. In this study, *P. americana*, *V. amygdalina*, and *C. olitorius* were the three plant species mostly used by traditional healers in the treatment of salmonelloses.

These results are similar to those reported by Agbankpè *et al*. [41] who documented that *V. amygdalina* is used like infusion to treat diarrheal infections in Cotonou and Abomey-Calavi (South-Benin), with a frequency relatively higher than the one of this study (16.80%).

The valorization of the 63 recorded plant species in this survey requires the research of their efficiency in further studies. A number of these plants have already been tested by different studies. Hounzangbé-Adoté *et al*. [36] showed that the seeds of *C. papaya* play an important role in the elimination of gastrointestinal worms in sheep and goats, by either killing the adult worms or by reducing the fertility of the females. Kermanshai *et al*. [66] demonstrated that benzyl isothiocyanate present in the seed of papaya is the compound responsible for its antihelminthic activity.

However, tannins and flavonoids could also exhibit this property. According to Hounzangbé-Adoté *et al*. [67] and Brunette and Hoste [68], tannins block *Haemonchus contortus* eggs from hatching, kill its adults and reduce their fertility. The frequent citation of *C. papaya* by several breeders is an evidence of confusion between salmonellosis and intestinal worms because they present mostly similar symptoms such as diarrhea. The research of antibacterial activities of this plant should be envisaged to verify whether is possesses antimicrobial properties against salmonelloses. Koffuor *et al*. [69] reported that *V. amygdalina* has strong antibacterial activity against salmonelloses. This is because they contain flavonoids, sesquiterpene lactones, saponins, tannins, alkaloids, and sterols in varying degrees [70-72].

Kpodekon *et al*. [35] demonstrated excellent antibacterial activities of the essential oil of *Ocimum gratissimum* on *Salmonella*, and this activity varies from one species to another. According to Kone [73], the leaves of *M. oleifera* have excellent antimicrobial activities because of their high content in sterols, triterpenes, and phenolic compounds notably tannins and flavonoids. These results show the enormous potentiality of the leaves of *M. oleifera*. The subsequent research of antibacterial properties of this plant against salmonelloses could be an alternative regarding issues of multidrug resistance.

The literature review revealed a number of plants that were not recorded in this study but known to have interesting anti-*Salmonella* properties. *Ocimum basilicum* for instance possesses an inhibitory activity on *Salmonella* Enterica [35]. This activity was confirmed by the chemical identification of pharmacological molecules such as estragole, linalool, and eugenol in that plant. Other studies reported the activity of the essential oils of *Salvia officinal* is and *Schinus molle* on *Salmonella* strains [48,49]. Keplinger *et al*. [53] described an anti-*S. Typhi* activity of *Uncaria tomentosa*. These properties are justified by the presence of pentacyclic and tetracyclics oxindole alkaloids in the organs of this plant. Many other plants were enumerated by different studies but without thorough pharmacological confirmations. All these data come to enrich the current database of Beninese plants that can be used in the treatment of salmonelloses.

Knowing that many people in Africa resort to herbal medicines as their first line of treatment when they are sick [74], the valorization of the recorded plant species through phytochemical and pharmacological studies could contribute to establishing evidence-based information on medicinal plants from African flora in general and Beninese in particular. It could be a contribution to the discovery of new molecules well controlled in terms of dosage to overcome challenges related to MDR *Salmonella* species that are hard to treat.

## Conclusion

This study conducted in Southern Benin confirms that veterinary pharmacopeia is still least practiced among animal producers especially those dealing with monogastrics such as poultry and pigs. On the other hand, traditional healers use a number of medicinal plants in the treatment of typhoid fever. The association of herbal medicine and modern medicine can lead to an effective control of salmonelloses in animals and human. It therefore urges, to envisage experimental studies to validate the effectiveness of the recipes obtained from this study for future use. At this point, it is important to restore a mutual confidence between traditional healers and scientists to organize them for the betterment of ancestral knowledge that they have in herbal medicine. This will be very helpful to circumvent cost-related challenges of antibiotics and the MDR occurrences leading to treatment failures in developing countries and the world as a whole.

## Authors’ Contributions

TVD, YMGH, JA, TJD, FG, MB, HSB and LB have designed the concept and supervised the plan of work. They have also prepared the questionnaires and have prepared the manuscript. ED, BL, AA, HK, KF, AAniambossou, PA, EH, MS, FA and LF helped in collecting data and provided technical support. TVD, YMGH, ED, LB and JA analyzed and interpreted the data. All authors read and approved the final manuscript.
